# Economics and outcomes of sotalol in‐patient dosing approaches in patients with atrial fibrillation

**DOI:** 10.1111/jce.15342

**Published:** 2022-01-05

**Authors:** Daniel L. Varela, Tyson S. Burnham, Heidi T. May, Tami L. Bair, Benjamin A. Steinberg, Joseph B. Muhlestein, Jeffrey L. Anderson, Kirk U. Knowlton, Thomas Jared Bunch

**Affiliations:** ^1^ Cardiology Division University of Utah School of Medicine Salt Lake City Utah USA; ^2^ Intermountain Medical Center Heart Institute Murray Utah USA

**Keywords:** antiarrhythmic drug loading, atrial fibrillation, cost economics, sotalol

## Abstract

**Introduction:**

There exists variability in the administration of in‐patient sotalol therapy for symptomatic atrial fibrillation (AF). The impact of this variability on patient in‐hospital and 30‐day posthospitalization costs and outcomes is not known. Also, the cost impact of intravenous sotalol, which can accelerate drug loading to therapeutic levels, is unknown.

**Methods:**

One hundred and thirty‐three AF patients admitted for oral sotalol initiation at an Intermountain Healthcare Hospital from January 2017 to December 2018 were included. Patient and dosing characteristics were described descriptively and the impact of dosing schedule was correlated with daily hospital costs/clinical outcomes during the index hospitalization and for 30 days. The Centers for Medicare and Medicaid Services reimbursement for 3‐day sotalol initiation is $9263.51. Projections of cost savings were made considering a 1‐day load using intravenous sotalol that costs $2500.00 to administer.

**Results:**

The average age was 70.3 ± 12.3 years and 60.2% were male with comorbidities of hypertension (83%), diabetes (36%), and coronary artery disease (53%). The mean ejection fraction was 59.9 ± 7.8% and the median corrected QT interval was 453.7 ± 37.6 ms before sotalol dosing. No ventricular arrhythmias developed, but bradycardia (<60 bpm) was observed in 37.6% of patients. The average length of stay was 3.9 ± 4.6 (median: 2.2) days. Postdischarge outcomes and rehospitalization rates stratified by length of stay were similar. The cost per day was estimated at $2931.55 (1. $2931.55, 2. $5863.10, 3. $8794.65, 4. $11 726.20).

**Conclusions:**

In‐patient oral sotalol dosing is markedly variable and results in the potential of both cost gain and loss to a hospital. In consideration of estimated costs, there is the potential for $871.55 cost savings compared to a 2‐day oral load and $3803.10 compared to a 3‐day oral load.

**Abbreviations**
AF: atrial fibrillation
AFL: atrial flutter
CMS: Centers for Medicare and Medicaid Service
ECG: electrocardiogram
ED: emergency department
FDA: Food and Drug Administration
IV: intravenous
VF: ventricular fibrillation
VT: ventricular tachycardia

## 1 BACKGROUND

Atrial fibrillation (AF) is the most common arrhythmia, with a lifetime incidence estimated at ~20%.^1^ The total number of cases of AF in the United States is expected to exceed 2.5 million by the year 2030 and 7.5 million by 2050.^1^, ^2^ AF carries significant morbidity and is one of the leading causes for hospitalization in the United States, with annual AF admissions exceeding 450 000 cases per year since 2010.^1^, ^2^ The cost burden of AF on the US Healthcare System is huge, with an estimated $10 billion spent managing AF in 2014.^3^ With increasing numbers of new AF cases each year, the financial burden imposed by AF continues to grow.

Antiarrhythmic drugs (AADs) still play a key role in the rhythm‐based management of AF; nonetheless, there remains a need to better understand protocols to administer these medications and monitor for side effects safely and effectively. The class III AAD sotalol is often loaded in the in‐patient setting, which requires several days of hospitalization for monitoring of drug side effects until steady state. In‐patient initiation of AAD therapy has been identified as a high‐cost burden aspect of AF‐related care.^4^ The cost associated with sotalol is particularly concerning, as it has risen considerably over the years and is only expected to continue to rise. A 3‐day hospitalization for sotalol initiation cost approximately $3500 in 2009 and increased to $10 000–$12 000 in 2019.^5^ The single greatest expense incurred during a patient's admission for sotalol initiation is room and board, with costs of approximately $2000–$3000 per night. Strategies aimed at reducing hospital length of stay may help drive down costs associated with sotalol initiation.

Despite a Food and Drug Administration (FDA) black box warning and societal recommendations that advocate for a 3‐day hospitalization and inpatient monitoring for QT prolongation and ventricular arrhythmias during the initiation of sotalol therapy, there exists considerable variability in how this drug is loaded in clinical practice.^6^ The impact of this variability on hospitalization costs and patient outcomes is unknown. One potential avenue for shortening a patient's length of stay would be to administer sotalol in a formulation that allows for more rapid achievement of steady‐state concentration, facilitating the earlier detection of potential side effects.

Intravenous (IV) sotalol was initially given FDA approval for use in 2010 as a substitute for oral therapy in patients unable to take medications orally. In March 2020, IV sotalol received FDA approval for use as part of the drug's initial loading protocol.^7^, ^8^ The IV administration shortens the time to steady‐state plasma concentration from 3 days to just 1–2 days (single IV dose plus 1–2 oral doses). The ability to reduce hospital length of stay could significantly reduce costs associated with in‐patient sotalol initiation and mitigate the overall economic burden that AF imposes on the healthcare system. Furthermore, protocol‐based IV sotalol administration may eliminate the dosing variability that currently exists in clinical practice and potentially imparts the risk of drug underdosing or overdosing.

This study aims to investigate the average length of stay for oral sotalol initiation admissions and evaluate the costs and clinical outcomes stratified according to initial hospitalization length of stay. Additionally, the study will quantify the sotalol dose at discharge to help provide clarity regarding potential underdosing or overdosing of drug therapy. Finally, this study will explore the cost impact of a 1‐day IV sotalol load to determine if there is a potential for cost savings that might be conferred by switching from an oral to IV sotalol loading protocol secondary to a shortened hospital stay.

## 2 METHODS

### 2.1 Study design and patient selection

A retrospective, single‐center study was performed among AF and atrial flutter (AFL) patients who were admitted to an Intermountain Healthcare hospital between January 1, 2017 and December 31, 2018, a time period to provide an understanding of more contemporary practices. Patients were included if they were at least 18 years of age, had an AF or AFL diagnosis, first AF or AFL hospitalization was between January 1, 2017 and December 31, 2018, and received at least two doses of oral sotalol during their index admission for sotalol initiation. Patients were excluded if they had ever been treated with sotalol or dofetilide before enrollment. Patients with congenital heart disease were excluded (see attached list of International Statistical Classification of Diseases and Related Health Problems (ICD) codes in Supporting information A). A total of 133 patients met the inclusion criteria.

### 2.2 Demographics, other risk factors, and clinical assessments

Patient demographics and clinical characteristics included age, sex, diabetes mellitus, hypertension, hyperlipidemia, smoking, heart failure, coronary artery disease (CAD), prior myocardial infarction, peripheral arterial disease, sleep apnea, dementia, cardiomyopathy, and prior malignancy (see Table 1 for complete list). Measurements of body mass index and ejection fraction were recorded. Use of prior medications and sotalol dosing were available. These variables were determined by electronic medical records and ICD‐ 9/10 codes that pre‐dated index hospitalization.

**Table 1:** Baseline characteristics, medications, and in‐hospital treatments and procedures

	Patients admitted for sotalol loading (*n* = 133)
*Characteristics and demographics*	
Age (years)	70.0 ± 12.3 (median: 71)
Sex (male)	80 (60.2%)
Insurance type	
Private	27 (20.3%)
Medicare	101 (75.9%)
Medicaid	3 (2.3%)
Self‐pay	2 (1.5%)
*Comorbidities and risk factors*	
Hypertension	110 (82.7%)
Hyperlipidemia	96 (72.2%)
Diabetes	48 (36.1%)
Past or current smoking	40 (30.1%)
History of depression	29 (21.8%)
Transient ischemic attack	10 (7.5%)
Stroke	17 (12.8%)
Myocardial infarction	18 (13.5%)
Coronary artery disease	71 (53.4%)
Peripheral arterial disease	6 (4.5%)
Chronic obstructive pulmonary disease	19 (14.3%)
Cardiomyopathy	43 (32.3%)
Dementia	4 (3.0%)
Sleep apnea	54 (40.6%)
Prior malignancy	17 (12.8%)
CHADS_2_	
Mean ± SD (median)	1.9 ± 1.2
0–1	59 (44.4%)
2–4	68 (51.1%)
≥5	6 (4.5%)
CHA_2_DS_2_‐VASc	
Mean ± SD (median)	5.0 ± 1.5
0–1	1 (0.8%)
2–4	49 (36.8%)
≥5	83 (62.4%)
EF (%)	59.9 ± 7.8 (median: 60)
BMI (kg/m^2^)	29.9 ± 7.8 (median: 29.9)
Creatinine	1.08 ± 0.45 (median: 0.97)
*Prior procedures*	
Prior ablation	27 (20.3%)
Prior cardioversion	43 (32.2%)
ICD	2 (1.5%)
*Hospitalization*	
Admitting arrhythmia	
Atrial fibrillation	125 (94.0%)
Atrial flutter	31 (23.3%)
Length of stay (days)	3.9 ± 4.6 (median: 2.2)
*In‐hospital medications received*	
Statin	76 (57.1%)
Calcium channel blocker	39 (29.3%)
ACE inhibitor	35 (26.3%)
ARB	21 (15.8%)
Diuretic	50 (37.6%)
Antiplatelet	53 (39.8%)
Warfarin	25 (18.8%)
Direct oral anticoagulant	73 (54.9%)
Antidepressant	30 (22.6%)

*Note*: Continuous data are presented as means ± standard deviation; categorical data are given as the counts (percentage).

Abbreviations: ACE, angiotensin‐converting enzyme; ARB, angiotensin receptor blocker; BMI, body mass index; CHADS_2_, congestive heart failure, hypertension, age >75, diabetes, stroke; CHA_2_DS_2_‐VASc, congestive heart failure, hypertension, age >75, diabetes, stroke, vascular disease, female gender; EF, ejection fraction; ICD, implantable cardiac defibrillator; IQR, interquartile range; SD, standard deviation.

### 2.3 Endpoints and outcome measures

In‐patient outcomes evaluated were those related to sotalol dosing, which included length of stay, electrocardiographic changes in response to sotalol initiation (increased Q wave, R wave and S wave (QRS) duration and QT/corrected QT interval (QTc) prolongation), the presence of significant bradycardia, stroke, and death. The postdischarge outcomes assessed were death, recurrent AF or AFL, stroke, ventricular tachycardia (VT), ventricular fibrillation (VF), Torsades de Pointes, QTc prolongation, bradycardia, emergency department (ED) visit, and any hospital readmission. Death was determined using electronic medical records and the state of Utah death certificates. Other outcomes were determined by electronic medical records and ICD‐9/10 codes. Arrhythmia‐related outcomes utilized the electrocardiogram (ECG) database, which includes ECGs, ambulatory monitors, and symptom‐ and auto‐triggered event monitors from all Intermountain Healthcare facilities. This database is updated daily with the completion of the dictated medical reports and physician review of the ordered ECGs.

Costs for in‐hospital oral sotalol initiation were determined using a combination of several sources. Data from the Cerner Health Facts EHR database between January 2009 and December 2017 were extracted and included to help determine the total number of AF and AFL annual caseloads. A budget impact model, which was modeled by Boston Strategic Partners, Inc., as contracted by AltaThera Pharmaceuticals, Inc., was also leveraged to identify the cost of room and board, administering medications, and patient monitoring.^9^ Within the budget impact model, the healthcare provider labor costs (physicians, nurses, and pharmacists) were derived from the Bureau of Labor Statistics. The drug costs were derived from Micromedix‐RED Book and the EKG costs were derived from Healthcare Blue Book. Other sources were used to define telemetry costs and room costs per day. The cost data as such are not specific to Intermountain Healthcare. Costs were determined by usual care (personnel, room, etc.) and also by the actual frequency of use by the patient (number of ECGs performed, etc). Hospitalization costs used the 3‐day Centers for Medicare and Medicaid Services (CMS) reimbursement for sotalol initiation of $9263.51. Projections of cost savings were made by comparing the cost of a 1‐day IV sotalol load of $2500.00 as projected by AltaThera.

### 2.4 Statistical analysis

The *χ* ^2^ statistic, Fisher's exact test, Student's *t *test, and analysis of variance were used to evaluate baseline and clinical characteristics among the patient groups. Initial evaluation to endpoints utilized the *χ* ^2^ statistic, the Fisher's exact test, and the Kaplan–Meier survival estimates and the log‐rank test. To confirm associations determined by univariable analysis, multivariable Cox hazard regression (SPSS, version 22.0) was performed to determine hazard ratios (HRs). Final models entered significant (*p* < .05) and confounding (10% change in HR) baseline covariables. Two‐tailed *p* values of ≤.05 were designated to be nominally significant.

## 3 RESULTS

### 3.1 Demographics

A total of 133 AF/AFL patients were admitted for sotalol initiation between January 1, 2017 and December 31, 2018 and met the study inclusion criteria. Baseline demographics and clinical characteristics are reported for the study population in Table 1. The average age was 70.0 ± 12.3 years and 60.2% were male. Noteworthy baseline comorbidities included hypertension (82.7%), hyperlipidemia (72.2%), diabetes (36.1%), CAD (53.4%), cardiomyopathy (32.3%), past or current smoking history (30.1%), and obstructive sleep apnea (40.6%). Mean baseline creatinine was 1.08 ± 0.45 (median: 0.97). Prior ablation occurred in 20.3% of patients and 32.3% had undergone cardioversion before enrollment. A total of 94.0% of patients were admitted with a primary diagnosis of AF and 23.2% with a diagnosis of AFL (with some patients having a prior diagnosis of both arrhythmias). Patients presented with a mean ejection fraction of 59.9 ± 7.8% and a mean QTc of 453.7 ± 37.6 ms before sotalol dosing; a baseline left bundle branch block was present in 4.5% and right bundle branch block in an additional 8.3% (Table 3).

### 3.2 Characteristics of index sotalol initiation admission

Sotalol was dosed twice daily based upon prescribing physician preferences, with ECGs performed routinely every 12 h (1–2 h after each dose of sotalol). Within the study population, 24 patients (18.1%) received only two doses, 53 (39.8%) received only three doses, 18 (13.5%) received only four doses, 13 (9.8%) received only five doses, and 25 (18.8%) received six doses or more (Table 2). The mean QTc before sotalol initiation was 453.7 ± 37.6 ms. After the first six doses of sotalol, the mean QTc of this population that remained on therapy was unchanged at 457.4 ± 37.6 ms (Table 3). The number of days spent in the hospital based upon twice daily in‐patient dosing was: 1 day (42.1%), 2 days (17.3%), 3 days (12.8%), ≥4 days (26.3%) (Table 4). On average, patients were in the hospital 3.9 ± 4.6 days (median: 2.2 days). In‐hospital outcomes included death: 2 (1.5%); stroke: 1 (0.8%); VT/VF: 0 (0%); and bradycardia (<50 bpm): 37 (27.8%).

**Table 2:** Sotalol dosing and dosage distributions

Number of doses, *n* (%)	
2	24 (18.1)
3	53 (39.8)
4	18 (13.5)
5	13 (9.8)
≥6	25 (18.8)
Minimum dosage per administration, *n* (%)	
40 mg	36 (27.1)
60 mg	3 (2.2)
80 mg	83 (62.4)
120 mg	10 (7.5)
Maximum dosage per administration, *n* (%)	
40 mg	28 (21.2)
60 mg	1 (0.8)
80 mg	96 (72.1)
120 mg	6 (4.5)
160 mg	1 (0.8)

**Table 3:** In‐hospital electrocardiographic trends in response to sotalol therapy

	Patients admitted for sotalol loading (*n* = 133)
*In‐hospital index ECG (before receiving sotalol)*	
PR	
Mean ± SD	179.8 ± 42.8
Median (IQR)	177 (152, 202)
QRS	
Mean ± SD	94.5 ± 22.0
Median (IQR)	88 (80, 100)
QTc	
Mean ± SD	453.7 ± 37.6
Median (IQR)	451 (430, 478)
RBBB	11 (8.3%)
LBBB	6 (4.5%)
*ECG obtained after the sixth dose of sotalol*	
PR	
Mean ± SD	167.6 ± 71.4
Median (IQR)	180 (153, 197)
QRS	
Mean ± SD	94.0 ± 11.2
Median (IQR)	92 (82.5, 105.5)
QTc	
Mean ± SD	457.4 ± 37.6
Median (IQR)	452 (414.5, 491)

Abbreviations: ECG, electrocardiogram; IQR, interquartile range; LBBB, left bundle branch block; QRS, Q wave, R wave and S wave; QTc, corrected QT interval; RBBB, right bundle branch block; SD, standard deviation.

**Table 4:** Frequency of outcomes (post hospitalization) stratified by length of stay categories for index sotalol initiation admission

	Total (*n* = 133)	1 day (*n* = 56)	2 days (*n* = 23)	3 days (*n* = 17)	≥4 days (*n* = 35)	*p* value
Death	13 (9.9%)	2 (3.6%)	4 (17.4%)	1 (5.9%)	6 (17.1%)	.0875
AF (per ECG)	46 (35.1%)	19 (33.9%)	7 (30.4%)	7 (41.2%)	13 (37.1%)	.90
AFL (per ECG)	33 (25.2%)	14 (25.0%)	4 (17.4%)	4 (23.5%)	11 (31.4%)	.70
Stroke	4 (3.1%)	2 (3.6%)	0 (0%)	1 (5.9%)	1 (2.9%)	.81
VT (per ECG)	0 (0%)	0 (0%)	0 (0%)	0 (0%)	0 (0%)	—
Torsades de Pointes (per ECG)	0 (0%)	0 (0%)	0 (0%)	0 (0%)	0 (0%)	—
VF (per ECG)	0 (0%)	0 (0%)	0 (0%)	0 (0%)	0 (0%)	—
QTc prolongation (>500)	38 (29.0%)	10 (17.9%)	5 (21.7%)	9 (52.9%)	14 (40.0%)	.02
Bradycardia (<60 bpm)	50 (38.2%)	26 (46.4%)	8 (34.8%)	6 (35.3%)	10 (28.6%)	.38
ED readmission						
30 days	7 (5.3%)	2 (3.6%)	2 (8.7%)	1 (5.9%)	2 (5.7%)	.74
60 days	15 (11.5%)	7 (12.5%)	3 (13.0%)	1 (5.9%)	4 (11.4%)	.96
90 days	17 (13.0%)	8 (14.3%)	3 (13.0%)	1 (5.9%)	5 (14.3%)	.91
Any hospital readmission						
30 days	10 (7.6%)	4 (7.1%)	2 (8.7%)	0 (0%)	4 (11.4%)	.61
60 days	18 (13.7%)	7 (12.5%)	4 (17.4%)	0 (0%)	7 (20.0%)	.22
90 days	24 (18.3%)	9 (16.1%)	5 (21.7%)	2 (11.8%)	8 (22.9%)	.75

Abbreviations: AF, atrial fibrillation; AFL, atrial flutter; ECG, electrocardiogram; ED, emergency department; QRS, Q wave, R wave and S wave; QTc, corrected QT interval; VF, ventricular fibrillation; VT, ventricular tachycardia.

*QTc prolongation is defined as a QTc >500 ms for patients with a QRS <120 ms or a QTc >550 ms for patients with a QRS >120 ms or ventricularly paced rhythm.

### 3.3 Posthospitalization outcomes

**Figure 1:**
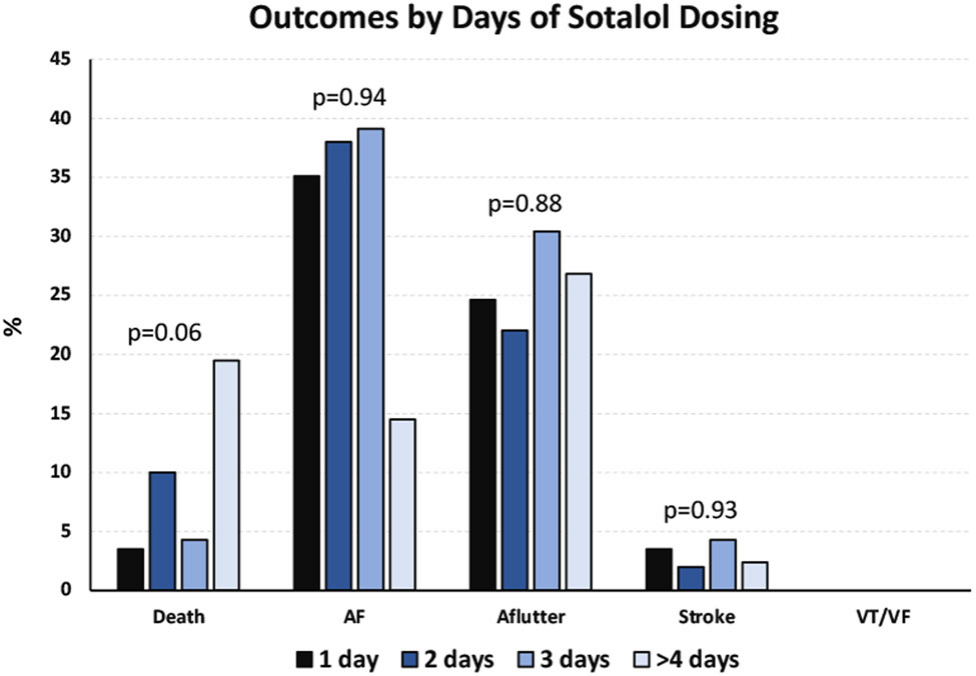
Cardiovascular outcomes are shown and compared based upon time of index in‐patient sotalol initiation. There were no significant differences in outcomes observed when comparing time‐based loading strategies. AF, atrial fibrillation; Aflutter, atrial flutter; VF, ventricular fibrillation; VT, ventricular tachycardia

The median length of follow‐up was 352 days, with those dying occurring at a median of 61 days (vs. 395 for those not dying, *p* < .0001). Recurrent atrial arrhythmia was the most common complication observed after discharge, with AF occurring in 35.1% of patients and AFL occurring in 25.2% (Table 4). A total of 13 patients (9.9%) died, 4 patients (3.1%) had a stroke and 38 patients (29.0%) experienced QTc prolongation (>500 ms in the absence of a bundle branch block and >550 ms in the presence of a bundle branch block). No patients suffered from any ventricular arrhythmias (VT, VF, or Torsades de Pointes) during the follow‐up period. A total of 50 patients (37.6%) experienced bradycardia (<60 bpm) by ECG. ED readmission rates were 5.3%, 11.5%, and 13.0% at 30, 60, and 90 days, respectively, while hospital readmission rates were 7.6%, 13.7%, and 18.3% at 30, 60, and 90 days (Figure 1).

**Figure 2:**
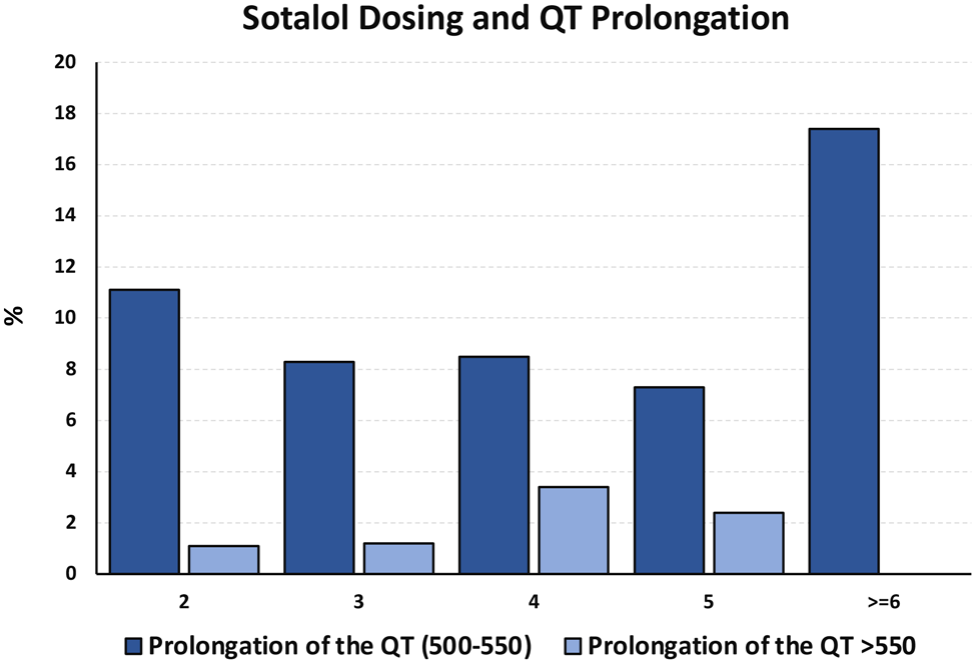
The percentage of patients who developed QT prolongation is shown and stratified by the number of doses of sotalol received during the index hospitalization. Patients with longer stays had higher rates of QT prolongation, which may be related to the stay and additional need for drug titration, as well as the use of sotalol in sick patients that also inherently required longer hospital stays

The rate of QTc prolongation (>500 ms) observed during follow‐up differed significantly based on the length of initial hospitalization. Postdischarge QTc prolongation occurred in 17.9% and 21.7% of patients initially admitted for 1 and 2 days, respectively, while it occurred in 52.9% of patients admitted for 3 days and 40.5% of patients who spent 4 or more days in the hospital during their index hospitalization for sotalol initiation, prompting dose titration (Figure 2). Death rates during follow‐up based upon time of hospitalization were: 3.6% for 1 day, 17.4% for 2 days, 5.9% for 3 days, and 17.1% for 4 or more days hospitalizations. These differences in death rates between groups did not achieve statistical significance (*p* = .08). The remaining posthospitalization outcomes, including recurrence of AF and AFL, bradycardia, stroke, and readmission rates, were similar regardless of the length of stay during index admission for sotalol initiation.

### 3.4 Cost economics of oral and IV sotalol initiation

The costs associated with a 3‐day admission for oral sotalol initiation are outlined in Table 5. The average cost per patient per day was $3611 ± $1049 (median: $3283). Average total cost per 3‐day admission was $12 466 ± $12 652 (median: $8569). The greatest contributor to the costs of hospitalization was room and board, with the hospital room itself being the single largest contributing factor ($1888.67 per day and $5666.01 per admission). The combined physician, nursing, and pharmacy labor costs also played a substantial role in the costs of hospitalization ($1462.66 per 3‐day admission). The cost of oral sotalol was minimal, at only $4.50 per tablet ($27 for the standard six doses administered during a 3‐day admission). The remaining costs of hospitalization were driven primarily by the costs of other medications (e.g., anticoagulation and saline), labs, and ECGs obtained while monitoring for side effects associated with sotalol. The CMS reimbursement for 3‐day oral sotalol initiation is $9263.51. In consideration of alternative approaches to sotalol in‐patient administration, IV sotalol is projected to cost $2500.00 per dose. However, IV sotalol loading has the potential to reduce hospital length of stay by one or more days. This translates to a projected cost savings of $871.55 when comparing a 1‐day IV sotalol load against a 2‐day oral load and $3803.10 when compared with a 3‐day oral load, due to reductions in hospital length of stay and a decrease in associated costs (e.g., daily room and board and provider labor costs).

**Table 5:** Hospitalization costs of care for oral sotalol initiation

	Cost per unit	Cost per day	Cost per 3‐day admission	Projected annual costs for in‐patient sotalol initiation (*n* = 133)
Medication costs				
Oral sotalol	$4.50	$9.00	$27.00	$3591.00
Anticoagulation		$100	$300	$39 900.00
IV saline		$6.92	$20.76	$2761.08
Labor costs				
Physician labor			$304.24	$40 463.92
Nurse labor			$947.40	$126 004.20
Pharmacy labor			$211.02	$28 065.66
Diagnostic testing costs				
Labs	$26.66		$111.96	$14 890.68
ECG	$127	$254	$762	$101 346
Room and board costs				
Hospital room		$1888.67	$5666.01	$753 579.33
Telemetry		$56.65	$169.95	$22 603.35
Miscellaneous equipment		$16.22	$48.66	$145.98
Total costs		$3611 ± $1049 (median: $3283)	$12 466 ± $12 652 (median: $8569)	Median: $1 139 677

Abbreviations: ECG electrocardiogram; IV, intravenous.

## 4 DISCUSSION

In the present study, we found significant variability in the in‐patient dosing regimens of oral sotalol during drug initiation, likely due to the inconsistent following of recommendations in actual clinical practice. Societal recommendations encourage the administration of 5–6 in‐patient doses of oral sotalol under close in‐patient monitoring; however, <40% of patients in our study received that many doses. Additionally, sotalol therapy at discharge in approximately one‐quarter of the population was suboptimal at 40 mg twice daily, a dose lower than the minimum recommended by the FDA, as the drug only exerts beta‐blocking effects without the Vaughan–Williams class III properties needed to exert antiarrhythmic (or QT‐prolonging) effects.^5^, ^8^, ^10^, ^11^, ^12^ As a consequence of this apparent drug underdosing, the very high rates of arrhythmia recurrence (approximately 1/3 of the population) are not surprising. Modeled use of IV sotalol would have resulted in cost savings by reducing the length of stay, and, when introduced into a pharmacokinetic‐guided protocol for administration, will likely minimize dosing variation and the potential for under‐ and overdosing of drug therapy.

In addition to the variability in drug dosing and the number of inpatient sotalol doses received, we found additional evidence of a deviation from FDA and societal recommendations as pertains to the initiation of sotalol in the setting of baseline QTc prolongation. Mean baseline QTc duration was 453 ms in this study, and FDA labeling specifies that QT must be <450 ms before drug initiation. Part of the decision to initiate sotalol in patients with borderline or mild QTc prolongation may have been due to discrepancies between automated ECG derived QTc measurements and manually calculated QTc measurements used by the prescribing physicians, which may have been shorter than that calculated by the ECG algorithm. In clinical practice, some physicians may rely on a more liberal baseline QTc, such as 500 ms, before initiating sotalol. While this practice deviates from FDA recommendations, it is common. Additionally, 12.8% of patients had an underlying bundle branch block, which lengthens the QT interval and complicates calculations of QTc; other methods exist for calculating the QTc in the setting of a bundle branch block, and many of these methods would allow for the use of sotalol despite a slightly prolonged QT interval, after factoring in the patient's QRS duration and extent of interventricular conduction delay.

We observed that the mean QTc duration at the time of admission did not increase in response to sotalol by the time of hospital discharge. Some of this is inherent to the drug being initiated during a hospitalization to monitor for QT prolongation, as those with significant prolongation will have the sotalol dosage decreased or have the drug stopped altogether as evidenced by the fact that QT prolongation was noted to occur in approximately one‐fifth of the patient population at some point during their hospital stay, but the average recorded QT interval following 6 oral sotalol doses was virtually unchanged from baseline. An alternative explanation for the lack of QT prolongation may stem from issues with drug underdosing, as prior studies have shown a linear relationship between sotalol drug concentration and extent of QTc prolongation, with the greatest QTc prolongation being achieved once the drug has reached steady‐state concentration.^10^, ^11^, ^12^ In the same way that sotalol underdosing might explain the higher rates of arrhythmia recurrence, drug underdosing could also explain the lack of any significant increase in QTc duration. Similarly, potential drug underdosing could also explain the lack of any ventricular arrhythmias during follow‐up. One prior study reported a 5.8% incidence of new or increased ventricular arrhythmias in response to sotalol initiation.^13^ In that study, the median initiation and discharge dose were both 160 mg, which when compared to the results of our study where only ~5% of patients received doses >80 mg and none developed ventricular arrhythmias, supports the presence of a dose‐dependent increase in arrhythmia risk and suggest that the apparent lack of ventricular arrhythmias in the present study could be the result of drug underdosing.

Bradycardia and QTc prolongation were the most common in‐patient side effects associated with sotalol initiation, occurring in approximately 20%–30% of patients during the index hospitalization. Despite the high rates of bradycardia and QTc prolongation, no ventricular arrhythmias occurred during hospitalization.

Postdischarge bradycardia and QTc prolongation were also common, occurring in 30%–40% of patients at 12‐month follow‐up. Long‐term treatment failure (recurrent AF or AFL) was also common, occurring in roughly one‐third of patients; the high rate of treatment failure may have been due to the fact that approximately one‐third of all patients were discharged on less than the minimum recommended dosage of sotalol. Serious adverse events were more common during the follow‐up period, with death occurring in 10% of patients and stroke occurring in 3%, which reflects in part the inherent cardiovascular risk factors in disease in many patients in which sotalol is used for AF.

The economics behind hospital length of stay and its effects on costs to the healthcare system have been well studied, with many strategies having been employed to shorten patients' length of stay and thereby reduce hospitalization costs. These strategies have been implemented across a spectrum of medical specialties, including cardiology. Elective cardiac procedures could benefit immensely from interventions that facilitate same‐day or short‐stay hospital discharge. In 2018, Amin et al.^14^ assessed the costs associated with same‐day discharge following elective percutaneous coronary interventions and found an average cost savings of $5128 per procedure, without any increase in adverse events in the postprocedural setting or during 12‐month follow‐up. Decreased costs from room and board and central supplies made up most costs saved with same‐day versus non‐same‐day discharge in this study. The impact of cost savings would also be subject to the status classification of the patient, such as in‐patient versus outpatient. The model used currently reflects in‐patient classification, which is consistent with the in‐hospital administration of an antiarrhythmic drug with inherent risks of proarrhythmia.

In 2019, Dahmane et al.^10^ conducted a cost‐minimization analysis from the health sector perspective using Monte Carlo simulations to assess the cost savings for accelerated IV sotalol loading compared with a typical 3‐day oral sotalol loading protocol. They found cost savings of $3123 with IV sotalol when administered over the course of a 2‐day hospitalization and $4820 when completed over the course of a single overnight stay, with the majority of their cost savings coming from room and board expenses and hospital supplies. They did note that some of their projected savings with the accelerated IV loading protocol were offset by the costs of IV sotalol, which is much more expensive than the oral formulation at their institution ($1400–$2264 per 150 mg vial of IV sotalol vs. $0.10 per 80 mg tablet of per os sotalol). Despite the high cost of IV sotalol, the potential cost savings from reducing admission time compared to prior studies with IV sotalol initiation has been estimated between $3000 and $4000 when compared with a 3‐day oral load.^1^, ^5^, ^8^

In the present study, we found the average cost per 3‐day admission for oral sotalol initiation was $12 466 (median: $8569), with the greatest expense coming from the hospital room itself (45%–66% of total hospitalization costs). Hospital room costs are so high that they markedly exceeded the costs of the physician, nursing, and pharmacy labor combined (average $5666 vs. $1462, over the course of a 3‐day admission).

The average cost of admission reported in our study is comparable with the national costs for a 3‐day sotalol admission reported by the CMS ($10 000–$12 000), and CMS reimbursement for a 3‐day admission is $9263.51. Average daily costs of admission were high, at $3611 (median: $3283). The daily and total costs of admission for oral sotalol initiation add up; if all 133 patients enrolled at our Intermountain Healthcare Hospital during the 2‐year study period underwent 3‐day oral sotalol loads, the total costs for this population alone would have exceeded $1 000 000. When considering all patients admitted for sotalol initiation across the United States, the costs of sotalol initiation easily eclipse a billion dollars on an annual basis. With daily costs exceeding $3000, the potential for cost savings could be huge for any measure that might help shorten the overall hospitalization length of stay.

### 4.1 In consideration of IV sotalol

We proposed a 1‐day IV sotalol loading protocol similar to that proposed by Somberg et al.,^11^ where an initial IV dose of sotalol was administered over 1 h, followed by the first oral dose 5 h after the infusion and a second oral dose 12 h after the first oral dose. Pharmacokinetic–pharmacodynamic models were used to ensure that peak sotalol plasma concentrations were achieved after each dose, thereby providing three separate opportunities to monitor for adverse drug effects at peak sotalol concentration over the course of a 24‐h hospitalization. We projected cost savings using this IV loading protocol to be approximately $3803 per admission. This is consistent with prior studies, which have reported estimated cost savings of approximately $3000–$4000 per admission if measures were taken to reduce hospitalization time for sotalol initiation.^1^, ^4^ Not only are our projected cost savings in line with what has been estimated in these previous studies but they are also similar to those reported in the pharmacoeconomic analysis conducted by Dahmane et al.,^10^ where the investigators found projected savings of $3123 for a 2‐day IV sotalol loading protocol and $4820 for a 1‐day loading protocol. The slight increase in cost savings seen with a 1‐day IV loading protocol in their study relative to ours may have been due in part to the lower estimated cost of IV sotalol used in their analysis ($1413 vs. $2500 per vial). In addition to the difference in projected IV sotalol costs between the two studies, our study differs from the one published by Dahmane's group^10^ because it includes a larger number of patients (133 vs. 35) and reports the real‐world costs of hospitalization in addition to simulated cost savings projected for an IV loading protocol. Additionally, our data on hospitalization costs comes coupled with important clinical outcomes of AF‐related morbidity and mortality, as discussed above.

In addition to the many benefits conferred by a decreased hospitalization time, IV sotalol loading may offer additional benefits over oral loading. Oral sotalol is often underdosed, as was observed in our study, where approximately one‐quarter of patients were discharged on doses of sotalol below the minimum dosage recommended by the FDA. IV administration of sotalol may improve the ability to achieve therapeutic steady‐state drug concentration, thereby increasing the drug's ability to exert its therapeutic effect in maintaining sinus rhythm. This IV dosing protocol allows for quicker and more precise selection of the optimal therapeutic dose, which translates into improved drug efficacy. Improved drug efficacy carries the potential for additional cost savings, as more effective maintenance of sinus rhythm may help prevent the recurrence of atrial arrhythmias and subsequently decrease hospital readmission rates and the need for additional expensive interventions, such as cardioversion, ablation, and repeat hospitalizations for AAD re‐loading. Additionally, there exists a potential for additional opportunity cost savings that comes from freeing up hospital beds and facilitating additional admissions. In the current healthcare climate, where hospitals are being met with increasingly large patient volumes and more limited availability of hospital beds, the potential opportunity cost savings could be very significant.

### 4.2 Study limitations

The superior cost economics of IV sotalol over oral sotalol should be considered hypothesis‐generating, as they are based on projected cost savings analyses. Prospective studies that implement the proposed IV sotalol loading protocols need to be performed to evaluate the real‐world cost economics of IV sotalol and obtain a better understanding of the IV formulation's impact on drug efficacy and clinical outcomes.

The current study also did not track certain outcomes that may have been helpful for assessing sotalol's efficacy and safety profile, such as the percentage of patients who required electrical cardioversion in addition to sotalol during index hospitalization and the rate of sotalol discontinuation throughout the follow‐up period. Data for readmission diagnoses were also lacking, making it impossible to determine whether readmissions were predominantly driven by recurrent arrhythmias, other cardiac comorbidities (e.g., acute coronary syndrome and heart failure), or unrelated noncardiac medical conditions.

Furthermore, EKG QT, and QTc intervals reported in this analysis were measured by computer automation as opposed to having a trained MD interpret the true QT and QTc intervals. This could lead to some inaccuracy in the reported QT/QTc values. As QT/QTc were used as parameters to make decisions regarding oral sotalol doses and loading, this could lead to some minor inaccuracies in the data.

The use of ICD‐9/10 codes is successful in their ability to capture data only as far as they are coded correctly. However, this is a commonly used method for capturing data in retrospective research, one that holds up when compared with adjudication by comprehensive medical record review.^15^, ^16^, ^17^

The FDA approval of IV sotalol is based on translational research models and has not been directly studied in humans, and there are concerns that IV administration of sotalol may overshoot serum drug concentrations and increase the risk of QTc prolongation and ventricular arrhythmias—risks that may not be accounted for in translational research models.^6^ Reassuringly, a meta‐analysis conducted to assess the risk of ventricular arrhythmias following the rapid administration of high‐dose IV sotalol (1.5 mg/kg or max 100 mg IV infused over <30 min) found a similar, if not slightly lower, risk of ventricular arrhythmias relative to oral sotalol.^18^ While these findings lend credibility to the observations of the safety of IV sotalol and the rapid achievement of a therapeutic steady‐state plasma drug concentration, caution must still be maintained as hospitals begin to adopt IV sotalol loading protocols in clinical practice. Physicians need to remain vigilant and closely monitor for arrhythmias and other serious potential side effects that could occur as a result of this new route of administration for the long‐standing and familiar AAD, sotalol.

### 4.3 Future directions

This study compared hospitalization costs that were derived using real‐world data from patients admitted for oral sotalol initiation against projected/theoretical cost savings that could be derived using a proposed IV sotalol loading protocol. The projected cost savings reported in the present study support efforts to implement an IV sotalol loading protocol, but real‐world data from patients treated with IV sotalol are still needed to determine whether proposed IV loading protocols can truly be completed over the course of a single overnight hospitalization and to confirm the clinical efficacy and safety of IV sotalol loading protocols.

Additionally, some centers, particularly those outside of the United States, have implemented novel protocols for outpatient sotalol loading that employ ambulatory cardiac monitoring (e.g., cardiac event monitors, or implantable electronic pacemaker or defibrillator data) to monitor for ECG abnormalities such as QT prolongation and ventricular ectopy in the outpatient setting while loading patients with sotalol.^19^ If these novel approaches for outpatient sotalol loading prove safe and effective, then they may provide viable and cost‐effective alternatives to both oral and IV inpatient loading protocols. Additional studies will be needed on both IV sotalol and these novel outpatient oral sotalol loading strategies to determine the feasibility, efficacy, safety, and costs associated with each to determine the optimal drug formulation and loading protocols for this medication.

## 5 CONCLUSIONS

Costs associated with the management of AF continue to rise as treatment options for this condition become increasingly complex. The costs incurred during in‐patient admission for AAD loading have rapidly increased over the years and represent a huge financial burden to our healthcare system, while the costs of AADs themselves have remained fairly stable. IV sotalol offers an opportunity to mitigate some of these costs by expediting the drug initiation process and facilitating a faster time to hospital discharge. In addition to the financial benefits of reducing hospitalization time, a shorter hospital stay may also decrease the risk of iatrogenic complications tied to hospitalization, may improve patient satisfaction and willingness to initiate AAD therapy, and, when introduced with a pharmacokinetic‐guided protocol for administration, may reduce variability in dosing and the potential for inadvertent under‐ or overdosing.

## Supporting information

Supporting information.

## Data Availability

The data that support the findings of this study are available by request from Intermountain Healthcare and for the economics analysis from Altathera. Restrictions apply to the availability of these data, which were used under permission from these organizations.

## References

[1] Sheikh A , Patel NJ , Nalluri N , et al. Trends in hospitalization for atrial fibrillation: epidemiology, cost, and implications for the future. Prog Cardiovasc Dis. 2015;58:105‐116. 26162957 10.1016/j.pcad.2015.07.002

[2] Kim MH , Johnston SS , Chu BC , Dalal MR , Schulman KL . Estimation of total incremental health care costs in patients with atrial fibrillation in the United States. Circ Cardiovasc Qual Outcomes. 2011;4:313‐320. 21540439 10.1161/CIRCOUTCOMES.110.958165

[3] Rozen G , Hosseini SM , Kaadan MI , et al. Emergency department visits for atrial fibrillation in the united states: trends in admission rates and economic burden from 2007 to 2014. J Am Heart Assoc. 2018;7:e009024. 30030215 10.1161/JAHA.118.009024 PMC6201465

[4] Kim MH , Klingman D , Lin J , Pathak P , Battleman DS . Cost of hospital admission for antiarrhythmic drug initiation in atrial fibrillation. Ann Pharmacother. 2009;43:840‐848. 19417111 10.1345/aph.1L698

[5] Von Bergen NH , Beshish AG , Maginot KR . Outpatient intravenous sotalol load to replace 3‐day admission oral sotalol load. HeartRhythm Case Rep. 2019;5:382‐383. 31341782 10.1016/j.hrcr.2019.04.005 PMC6630186

[6] Biswas M , Levy A , Weber R , et al. Multicenter analysis of dosing protocols for sotalol initiation. J Cardiovasc Pharmacol Ther. 2020;25:212‐218. 31707834 10.1177/1074248419887710 PMC7113108

[7] Samanta R , Thiagalingam A , Turner C , Lakkireddy DJ , Kovoor P . The use of intravenous sotalol in cardiac arrhythmias. Heart Lung Circ. 2018;2018(27):1318‐1326. 10.1016/j.hlc.2018.03.017 29853342

[8] United States Food and Drug Administration and ALTATHERA Pharmaceuticals, L. C . Sotalol hydrochloride FDA label: highlights of prescribing information. Accessed November 2, 2020. https://www.accessdata.fda.gov/drugsatfda_docs/label//022306s005lblrpl.pdf

[9] Badani H , Seo BW , Peyerl FW . Pharmacoeconomic analysis of sotalol loading. Comparison of 1‐day inpatient loading of IV sotalol compared to standard 3 day loading protocol. Value Health. 2019;22:S122.

[10] Dahmane E , Tang K , Gobburu JVS , et al. Clinical pharmacology‐driven translational research to optimize bedside therapeutics of sotalol therapy. Clin Transl Sci. 2019;2019(12):648‐656. 10.1111/cts.12670 PMC6853149 31328888

[11] Somberg JC , Vinks AA , Dong M , Molnar J . Model‐informed development of sotalol loading and dose escalation employing an intravenous infusion. Cardiol Res. 2020;11:294‐304. 32849964 10.14740/cr1143 PMC7430892

[12] US FDA . US Food and Drug Administration (FDA) clinical pharmacology review—Betapace. Accessed December 6, 2020. https://www.accessdata.fda.gov/drugsatfda_docs/nda/2001/19865S010_Betapace_biopharmr.pdf

[13] Chung MK , Schweikert RA , Wilkoff BL , et al. Is hospital admission for initiation of antiarrhythmic therapy with sotalol for atrial arrhythmias required? Yield of in‐hospital monitoring and prediction of risk for significant arrhythmia complications. J Am Coll Cardiol. 1998;32:169‐176. 9669266 10.1016/s0735-1097(98)00189-2

[14] Amin AP , Pinto D , House JA , et al. Association of same‐day discharge after elective percutaneous coronary intervention in the United States with costs and outcomes. JAMA Cardiol. 2018;3:1041‐1049. 30267035 10.1001/jamacardio.2018.3029 PMC6583057

[15] Dewland TA , Glidden DV , Marcus GM . Healthcare utilization and clinical outcomes after catheter ablation of atrial flutter. PLoS ONE. 2014;9:e100509. 24983868 10.1371/journal.pone.0100509 PMC4077565

[16] Kim EJ , Hoffmann TJ , Nah G , Vittinghoff E , Delling F , Marcus GM . Coffee consumption and incident tachyarrhythmias: reported behavior, Mendelian randomization, and their interactions. JAMA Intern Med. 2021;181:1185‐1193. 34279564 10.1001/jamainternmed.2021.3616 PMC8290332

[17] Whitman IR , Agarwal V , Nah G , et al. Alcohol abuse and cardiac disease. J Am Coll Cardiol. 2017;69:13‐24. 28057245 10.1016/j.jacc.2016.10.048 PMC5226115

[18] Marill KA , Runge T . Meta‐analysis of the Risk of Torsades de Pointes in patients treated with intravenous racemic sotalol. Acad Emerg Med. 2001;8:117‐124. 11157286 10.1111/j.1553-2712.2001.tb01275.x

[19] Mascarenhas DAN , Mudumbi PC , Kantharia BK . Outpatient initiation of sotalol in patients with atrial fibrillation: utility of cardiac implantable electronic devices for therapy monitoring. Am J Cardiovasc Drugs. 2021;21:693‐700. 34291437 10.1007/s40256-021-00493-7 PMC8295005

