# Supplementary material for: Economics and outcomes of sotalol in‐patient dosing approaches in patients with atrial fibrillation
Source: J Cardiovasc Electrophysiol. 2022 Jan 5;33(3):333–42. doi: 10.1111/jce.15342 (PMC9305518; doi:10.1111/jce.15342)
Supplement: Supplementary file 1 — Supporting information. [file JCE-33-333-s001.docx]

**Supplement A**. Diagnosis-Related Group Codes

|  | **Patients Admitted for Sotalol Loading** (n=133) |
| --- | --- |
| 25 | 1 (0.8%) |
| 38 | 2 (1.5%) |
| 57 | 1 (0.8%) |
| 64 | 3 (2.3%) |
| 65 | 2 (1.5%) |
| 66 | 2 (1.5%) |
| 149 | 1 (0.8%) |
| 176 | 1 (0.8%) |
| 178 | 1 (0.8%) |
| 183 | 1 (0.8%) |
| 189 | 3 (2.3%) |
| 194 | 2 (1.5%) |
| 208 | 1 (0.8%) |
| 216 | 1 (0.8%) |
| 227 | 1 (0.8%) |
| 233 | 1 (0.8%) |
| 234 | 0 (0%) |
| 235 | 0 (0%) |
| 240 | 1 (0.8%) |
| 242 | 1 (0.8%) |
| 243 | 2 (1.5%) |
| 244 | 2 (1.5%) |
| 247 | 2 (1.5%) |
| 251 | 1 (0.8%) |
| 274 | 5 (3.8%) |
| 282 | 1 (0.8%) |
| 287 | 3 (2.3%) |
| 301 | 1 (0.8%) |
| 308 | 2 (1.5%) |
| 309 | 13 (9.8%) |
| 310 | 35 (26.3%) |
| 313 | 0 (0%) |
| 314 | 1 (0.8%) |
| 371 | 1 (0.8%) |
| 378 | 1 (0.8%) |
| 379 | 1 (0.8%) |
| 389 | 1 (0.8%) |
| 418 | 1 (0.8%) |
| 441 | 1 (0.8%) |
| 454 | 1 (0.8%) |
| 466 | 1 (0.8%) |
| 468 | 1 (0.8%) |
| 469 | 1 (0.8%) |
| 470 | 3 (2.3%) |
| 473 | 1 (0.8%) |
| 481 | 1 (0.8%) |
| 556 | 1 (0.8%) |
| 603 | 1 (0.8%) |
| 606 | 1 (0.8%) |
| 638 | 1 (0.8%) |
| 640 | 1 (0.8%) |
| 781 | 1 (0.8%) |
| 809 | 1 (0.8%) |
| 813 | 1 (0.8%) |
| 821 | 1 (0.8%) |
| 853 | 1 (0.8%) |
| 870 | 1 (0.8%) |
| 871 | 7 (5.3%) |
| 872 | 4 (3.0%) |
| 945 | 1 (0.8%) |
| 958 | 1 (0.8%) |
